# Gastroduodenal Obstruction Secondary to Pica-associated Bezoar: A Case Report

**DOI:** 10.5811/cpcem.21300

**Published:** 2025-01-06

**Authors:** Mariam Attia, Ashley A. Lavoie-Forrest, Phoebe Langius, Leon Melnitsky, Sandra Lopez, Eric Boccio

**Affiliations:** * Memorial Healthcare System, Department of Emergency Medicine, Hollywood, Florida; † Nova Southeastern University, Dr. Kiran C. Patel College of Osteopathic Medicine, Fort Lauderdale, Florida; ‡ Florida International University, Herbert Wertheim College of Medicine, Miami, Florida

**Keywords:** gastroduodenal obstruction, gastroduodenal bezoar, pica, iron-deficiency anemia, case report

## Abstract

**Introduction:**

While mild or moderate iron-deficiency anemia may not cause any symptoms, more severe deficiencies may present clinically as fatigue, shortness of breath, exertional dyspnea, lightheadedness, tachycardia, and presyncope or syncope, and, in rare instances, pica. Pica is defined as the developmentally inappropriate ingestion of non-nutritive, non-food substances for more than one month. We present the case of a duodenal obstruction secondary to a pica-associated bezoar in a patient with iron-deficiency anemia who presented to the emergency department (ED) with abdominal pain.

**Case Report:**

A 40-year-old female with past medical history of iron-deficiency anemia, asthma, and Von Willebrand disease and allergies to both oral and intravenous (IV) iron presented to the ED with one day of acute and severe abdominal pain associated with nausea and vomiting. The patient’s last bowel movement was one day prior to presentation. The abdominal exam revealed mild distention and generalized tenderness with no evidence of rebound or guarding. Computed tomography of the abdomen and pelvis with IV and oral contrast demonstrated gastric distention and a fecalized distal duodenum with wall thickening concerning for a duodenal obstruction. Given the patient’s known history of iron-deficiency anemia, the emergency physician inquired about ingestion of non-nutritive substances to which the patient replied that she had been consuming cotton foam. The patient was admitted to the hospital for gastroenterology consultation and esophagogastroduodenoscopy.

**Conclusion:**

Pica-associated gastrointestinal bezoars are a rare complication with a variety of reported substances being consumed. Patients presenting with small gastroduodenal bezoars may benefit from endoscopic removal, but large non-fragmentable bezoars can only be removed through surgical intervention.

## INTRODUCTION

While mild or moderate iron-deficiency anemia may not cause any symptoms, more severe deficiencies may present clinically as fatigue, shortness of breath, exertional dyspnea, lightheadedness, tachycardia, and presyncope or syncope. In rare instances, pica, or the developmentally inappropriate ingestion of non-food substances for more than one month, may be observed. Features of pica include the compulsory consumption of a variety of both organic and inorganic, digestible and indigestible, non-nutritive substances including ice, soil, clay, paper, hair, and cotton foam. While evidence suggests a strong association between pica and iron-deficiency anemia which is strengthened by the observation that pica improves or resolves following iron transfusions, the pathophysiology of pica is currently unknown. We present the case of a duodenal obstruction secondary to a pica-associated bezoar in a patient with iron-deficiency anemia who presented to the emergency department (ED) with abdominal pain.

## CASE REPORT

A 40-year-old female with past medical history of iron-deficiency anemia, asthma, and Von Willebrand disease, past surgical history of Cesarean section, and abdominal gunshot wound status post laparotomy (2019), and allergies to ferrous sulfate, iron dextran, and iron sucrose presented to the ED with one month of intermittent abdominal pain. The patient reported one day of acutely worsened and severe abdominal pain associated with nausea, non-bloody and nonbilious vomiting, and inability to pass flatus. The patient’s last bowel movement was one day prior to presentation and was described as grossly normal. The patient denied urinary complaints, fever, shaking, and chills. Initial vital signs including blood pressure (110/71 millimeters of mercury), heart rate (67 beats per minute), respiratory rate (18 breaths per minute), peripheral capillary oxygen saturation (100%, room air), and temperature (36.5 °Celsius, oral) were within normal limits.

**Image 1:**
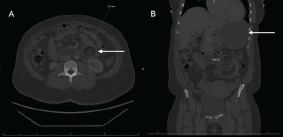
(A) Axial and (B) sagittal views of computed tomography of abdomen and pelvis with intravenous and oral contrast demonstrated gastric and duodenal distention (arrows) concerning for distal duodenal obstruction.

The abdominal exam revealed mild distention and generalized tenderness with no evidence of rebound or guarding. The points of maximal abdominal tenderness were the left upper quadrant and middle of the epigastric region. Murphy’s sign was not present. There were no visible or palpable abdominal masses. The complete blood count was significant for a microcytic anemia (hemoglobin 8.9 grams per deciliter (g/dL) [reference range 12.0–15.5 g/dL], hematocrit 32.1% [36–44%], and mean corpuscular volume 62.9 femtoliters (fL) [80–95 fL]). The comprehensive metabolic panel demonstrated hypokalemia (potassium 3.4 millimoles per liter (mmol/L) [3.5–5.5 mmol/L]). Iron level and transferrin saturation were low (25.0 micrograms per deciliter (mcg/dL) [60–170 mcg/dL] and 6% [20–50%], respectively). Zinc and calcium levels were not measured. Computed tomography of the abdomen and pelvis with intravenous (IV) and oral contrast demonstrated gastric distention and a distended fecalized distal duodenum with wall thickening, adjacent fat stranding, and prominence of vasa recta concerning for a duodenal obstruction; duodenal bezoar could not be excluded (Image 1).

**Image 2:**
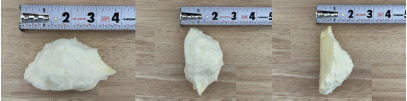
Piece of cotton foam (9.5 x 5.1 x 5.1 centimeter) presented to clinical team by patient after being asked about ingestion of non-nutritive substances. The patient stated that she had last ingested cotton foam two days prior to her presentation to the emergency department.

The patient received 6 milligrams (mg) of morphine IV, 4 mg ondansetron IV, 40 milliequivalents of potassium chloride IV over four hours, and 1 L of sodium chloride IV over one hour. The patient could not receive iron supplementation due to documented allergies. Given the patient’s known history of iron-deficiency anemia, the emergency physician inquired about ingestion of non-nutritive substances to which the patient replied that she had been consuming cotton foam since 2012 with last ingestion two days earlier. The patient appeared to have insight that eating cotton foam was illogical but could not verbalize the rationale behind her indulgence. The patient presented a large piece of cotton foam from her purse to show the clinical team (Image 2).

**CPC-EM Capsule**

What do we already know about this clinical entity?

*Severe deficiencies in iron may present clinically as fatigue, shortness of breath, tachycardia and, in rare instances, pica*.

What makes this presentation of disease reportable?

*Pica-associated gastrointestinal bezoars are a rare complication with a variety of non-nutritive consumed substances having been reported*.

What is the major learning point?

*It is important to inquire about dietary behaviors and frequency of non-nutritive substance ingestion when clinical suspicion for pica is high*.

How might this improve emergency medicine practice?

*When diagnosing a gastrointestinal bezoar in the severely iron-deficient patient, consider pica-related behaviors*.

The cotton foam was confiscated, and the patient was made nil per os and admitted to the hospital for gastroenterology consultation and esophagogastroduodenoscopy for duodenal obstruction secondary to pica-associated bezoar, presumably cotton foam.

## DISCUSSION

Anemia is a common clinical disorder affecting 1.92 billion people globally as of 2021.^1^ According to the National Hospital Ambulatory Medical Care Survey reported by the US Centers for Disease Control and Prevention, 800,000 cases of anemia were diagnosed in EDs across the United States in 2021, and iron-deficiency was the leading cause.^2^ While mild or moderate iron-deficiency anemia may not cause any symptoms, more severe deficiencies may present clinically as fatigue, shortness of breath, exertional dyspnea, lightheadedness, tachycardia, presyncope or syncope, and, in rare instances, pica. Pica is defined by the *Diagnostic and Statistical Manual of Mental Disorders*, 5^th^ edition, as developmentally inappropriate ingestion of non-nutritive, non-food substances for more than one month.^3^,^4^ It is theorized that pica is a compulsory and compensatory behavior caused by low nutritional status, specifically in iron, zinc, and calcium.^5^ Pica can be further categorized by the type of non-nutritive non-food substance being ingested. In the US, pagophagia, the ingestion of ice, is most common among patients with iron-deficiency. In other parts of the world and especially in Africa, geophagia, the ingestion of earth materials such as soil, dirt, or clay, is both common and socially accepted.^6^ The ingestion of hair is referred to as trichophagia.

Studies have shown an increased prevalence of pica among children and pregnant women. It is speculated that pica behaviors may develop more frequently in these subgroups due to a negative nutrient state exacerbated by high energy expenditures and increased nutritional needs.^7^ One study found that over half of pregnant adolescents (<18 years) reported pica behaviors, with pagophagia being the most common. These subjects had a significantly lower iron status than those who did not engage in pica behaviors.^5^ A study of 262 non-pregnant adults with iron-deficiency anemia found that 87.3% reported pagophagia.^8^ A meta-analysis of 43 studies involving 6,407 subjects found that individuals with pica were 2.35 times more likely to be anemic and have lower than normal zinc levels.^9^

Adults with avoidant or restrictive food intake disorder, poor body image, and depression are more likely to exhibit pica than the general population.^10^ A study of blood donors found an association between pica and low ferritin levels, non-Asian race, younger age, and restless leg syndrome with no statistically significant difference between the sexes.^11^ A recent case-control study found an increased prevalence of pica in individuals with autism spectrum disorder, intellectual disability, and developmental disabilities.^12^ Individuals demonstrated behaviors of chewing on inedible materials without swallowing prior to a formal diagnosis of pica being made.

Pica is an important disorder to identify, however, it is commonly overlooked due to patients’ unwillingness to speak about unusual eating behaviors and physicians’ failure to ask targeted questions. When clinical suspicion is high, the medical history should include direct questions about dietary behaviors, cravings, and frequency of non-nutritive substance ingestion. On physical examination, signs of nutritional deficiencies such as pallor and glossitis may be noted. While there are no specific screening or diagnostic tests for pica, serum laboratory tests including a complete blood count, serum iron level, iron saturation, total iron-binding capacity, unsaturated iron-binding capacity, and ferritin level should be considered. Results will most likely demonstrate a microcytic anemia with elevated, unsaturated iron-binding capacity and total iron-binding capacity and decreased transferrin levels.^13^ While serum ferritin is typically low, normal levels have been documented in pica cases.^11^

Therapeutic management of pica involves addressing the underlying cause of anemia and repleting iron and electrolytes when deficient. Treatments include oral and IV iron supplementation and packed red blood cell transfusions. In patients with comorbid iron-deficiency anemia, pica symptoms have been shown to resolve within three weeks following IV iron infusion.^6^ Additionally, prognosis has been shown to improve when psychiatric care, which includes cognitive behavioral therapy focused on controlling urges and discouraging compulsory ingestion of non-nutritive substances, is maintained.^14^ Documented allergies to oral and IV forms of iron made this case particularly interesting and the therapeutic management of the iron-deficiency anemia particularly challenging. Long-term management goals would need to focus on improving hemoglobin levels and reducing anemia-associated symptoms. Several treatment options include transfusion of packed red blood cells, administration of epoetin alfa, and desensitization treatment to oral and IV iron.^15^

Early recognition of pica can lead to better prevention of life-threatening complications such as infection, lead poisoning, gastrointestinal (GI) inflammation secondary to the ingestion of caustic chemicals, and GI obstruction secondary to bezoars.^16^ Bezoar refers to a mass of indigestible material that forms in the GI tract and may consist of organic or inorganic materials. Often, bezoars may cause GI obstruction, which presents clinically as severe abdominal pain, abdominal distention, nausea, vomiting, constipation, and inability to pass flatus. The workup of patients presenting with signs or symptoms of GI obstruction may include diagnostic imaging studies such as abdominal radiographs or computed tomography. Patients presenting with small gastric bezoars may benefit from endoscopic removal, but large non-fragmentable bezoars can only be removed through surgical intervention via laparotomy or laparoscopic extraction.^14^

## CONCLUSION

We present the case of a 40-year-old woman with past medical history of iron-deficiency anemia, and with allergies to both oral and IV iron, suffering from abdominal pain secondary to a duodenal obstruction caused by a pica-associated bezoar, presumably cotton foam. Pica-associated gastrointestinal bezoars are a rare complication with a variety of reported substances being consumed, including cotton foam, dirt, paper, and hair. Patients presenting with small gastroduodenal bezoars may benefit from endoscopic removal, but large non-fragmentable bezoars can only be removed through surgical intervention. The clinical workup for pica should include an assessment for iron-deficiency anemia and calcium and zinc levels. Therapeutic management is mostly supportive and should focus on iron repletion, correction of electrolyte abnormalities, and outpatient referral for scheduled iron transfusions and cognitive behavioral therapy.
